# COVID-19 Cases, Hospitalizations, and Deaths in Nursing Homes: Factors Impacting the Second Surge

**DOI:** 10.1093/geroni/igab046.056

**Published:** 2021-12-17

**Authors:** Sean Fleming, Danya Qato, Alexandra Wallem, Paulina Kepczynska, Linda Wastila, Tham Le, Jeanne Yang

**Affiliations:** 1 University of Maryland Baltimore, University of Maryland Baltimore, Maryland, United States; 2 University of Maryland School of Pharmacy, Baltimore, Maryland, United States

## Abstract

As of March 2021, over 128,000 nursing home (NH) residents have died due to COVID-19 complications, accounting for one-third of all U.S. COVID-19 deaths. Early studies highlighted factors which heightened residents’ risk—facility size and profit status, CMS Five-Star quality rating, race, and high Medicaid share. Despite improved nationwide social distancing and access to protective equipment, between October-December 2020 nursing home cases, hospitalizations, and deaths peaked to highest levels since the pandemic’s advent. The purpose of this study is to quantify previously unexamined associations between resident, facility, and geographic characteristics and COVID-19 infections, hospitalizations, and fatalities in nursing homes during this second surge. In this cross-sectional study, we constructed a novel dataset with linked facility- and county-level data from the CMS Nursing Home COVID-19 Public File, Nursing Home Compare, Long-Term Care Focus, and The New York Times. Multivariable logistic regression evaluated the odds of COVID-19 infections, hospitalizations, and deaths in nursing homes. Among 13,156 nursing homes, 80.5% reported ≥1 COVID-19 cases; on average, nursing homes reported 4.5 hospitalizations and 3.0 deaths. Facilities with higher acuity patients, chain status, >150 beds, high percentage white residents, low Medicaid share, high surrounding county case rates, and occupancy rates >75% were significantly (p <.001) related to increased odds of all outcomes. N95 mask shortages continued to increase risk of cases and hospitalizations. Five-Star ratings, high influenza vaccination rates, and clinical staff shortages were not significant factors. Findings demonstrate that through 2020, nursing homes continued to face challenges protecting their residents from COVID-19-related morbidity and mortality.

